# Prevalence of Head and Neck Sarcoma in a Major Cancer Center in Iran- A 10-Year Study

**Published:** 2019-03

**Authors:** Saede Atarbashi-Moghadam, Amir Nader Emami Razavi, Saman Salehi Zalani

**Affiliations:** 1 *Department of Oral and Maxillofacial Pathology, Dental School of Shahid Beheshti University, Tehran, Iran.*; 2 *PhD Iran National Tumor Bank, Cancer Institute, Tehran University of Medical Sciences, Tehran, Iran.*; 3 *Research Committee, Dental School of Shahid Beheshti University of Medical Sciences, Tehran, Iran.*

**Keywords:** Cancer of head and neck, Dermatofibrosarcoma, Osteosarcoma, Soft tissue sarcoma.

## Abstract

**Introduction:**

Sarcomas are rare malignancies with aggressive biological behavior. They are categorized into soft and hard tissue types. The main objective of this study was to analyze the prevalence of head and neck sarcomas (HNS) among the Iranian population.

**Materials and Methods:**

The pathology files derived from Iran National Tumor Bank of Cancer Institute in Imam Khomeini Hospital, affiliated to Tehran University of Medical Sciences, Tehran, Iran, served as the source of the materials for this study. All cases diagnosed with head and neck sarcoma were included in the study. The recorded data included the patient’s age, gender, tumor location, and rates of recurrence and metastasis.

**Results:**

Investigation of the pathology files of the patients referring to the center under study during a 10-year period resulted in the identification of 183 HNS cases, 96.17% of which were primary. Generally, the prevalence of this disease was at its highest level in patients within the age range of 30-60 years with a male to female ratio of 1.4. The recurrence and metastasis rates of HNS were 32.38% and 5%, respectively. Osteosarcoma was detected as the most common type of sarcoma. Soft tissue sarcomas constituted 69.3% of the lesions with a male predilection. The patients afflicted with this type of sarcoma had a mean age of 45.88 years. Furthermore, hard tissue sarcomas comprised 30.68% of the sarcoma cases with a mean age of 36.22 years and a female predilection. The commonest lesion was osteosarcoma, and the most typical location was the mandible.

**Conclusion:**

In the current study, head and neck sarcomas were most often observed in patients within the age range of 30-60 years with a male predilection. Osteosarcoma was identified as the most common type of sarcoma. Studies addressing rare lesions with a large sample size facilitate the recognition of the demographic data and histopathologic variation which may contribute to a correct diagnosis.

## Introduction

Sarcomas are a heterogeneous group of malignancies originating from the transformed cells of mesenchymal lineage with a broad range of microscopic types (1). They are usually divided into two groups of hard tissue (bone) sarcomas (HTSs) and soft tissue sarcomas (STSs), accounting for 20% and 80% of cases, respectively (1,2). These tumors are quite rare in the head and neck regions with a prevalence range of 4-10%; accordingly, they are less common than carcinomas (3,4). Surgery with wide resection margins is the treatment of choice for this disease, and radiation is frequently used as adjuvant therapy (5). 

In general, epidemiological studies with a large series have revealed valuable information on the prevalence and clinicopathological characteristics of the lesions in different countries (4). Therefore, the purpose of this study was to assess the pattern of head and neck sarcomas (HNSs) in one of the major pathology centers in Tehran, Iran, and compare these findings with those previously reported in other countries. 

## Materials and Methods

A retrospective, descriptive, cross-sectional study was performed using the pathology files of the Iran National Tumor Bank of Cancer Institute in Imam Khomeini Hospital affiliated to Tehran University of Medical Sciences, Tehran, with legal permission. The files were dated between the earliest time of their documentation in 2007 up to 2016. For the purpose of the study, the pathology reports of the files indicating HNS diagnosis were reviewed. The head and neck regions in this study were considered as any sites above the clavicles with the exception of the cerebrum and eyes. Moreover, the age and gender of the patients, location of the lesions, as well as recurrence and metastases rates were recorded and classified in tables. The data were analyzed in SPSS software (version 21) using descriptive statistics.

## Results

During the 10-year period investigated in this study, a total of 183 cases of HNSs had been registered in the center under study. The HNSs accounted for 4.7% of the sarcoma cases. Furthermore, 3.82% (n=7) and 96.17% (n=176) of the cases were metastatic and primary sarcomas, respectively (Table.1). This disease generally had its highest prevalence in patients within the age range of 30-60 years. The patients had the mean age of 42.92 years (age range: 3-86 years). The HSN cases consisted of 103 males (58.5%) and 73 females (41.4%), rendering a male to female ratio of 1.4. 

In total, 32.38% (n=57) of the patients presented recurrence, and about 5% (n=8) of them had metastasis. Osteosarcoma was the most prevalent sarcoma (25%), and oral cavity (37.5%) was the most commonly involved location. The HNSs were categorized into two groups of STS and HTS.

**Table 1:** Demographic data of the patients with metastatic sarcoma of the head and neck

**Histologic type **	**Gender **	**Age (year)**	**Site of metastasis**	**Primary location**
Metastatic spindle cell sarcoma	Female	27	Cervical lymph node	Uterine
	Male	80	Skull	Unknown
Metastatic synovial sarcoma	Male	41	Scalp	Elbow
Metastatic Ewing sarcoma	Male	17	Scalp	Unknown
Metastatic angiosarcoma	Male	44	Cervical lymph node	Unknown
Metastatic chondrosarcoma	Male	27	Cervical lymph node	Scapula
Metastatic high-grade sarcoma	Male	24	Cervical lymph node	Unknown
				
				

*Soft tissue sarcoma*

Soft tissue sarcoma constituted 69.31% (n=122) of the lesions. Fourteen different microscopic types of soft tissue sarcomas had been diagnosed in the patients under study. The most common histopathological variants were unclassified sarcomas(e.g.,spindle,pleomorphic, and undifferentiated sarcomas; 21%), dermato- fibrosarcoma (12.5%), and rhabdomyosarcoma (9.65%). Face/scalp (43.36%) and neck (21.31%) were the most commonly affected locations, followed by the oral cavity (18.85%) and sinonasal region (9.01%). The STS had a male to female ratio of 1.77. The prevalence of this type of sarcoma was at its highest level in patients within the age range of 30-60 with a mean age of 45.88 years (Table.2). 

**Table 2:** Distribution of primary soft tissue sarcoma cases based on age, gender, recurrence, and metastasis (n=122)

**Histopathologic type**	**Number of cases**	**Age**			**Gender**		**Recurrence (n)**	**Metastasis (n)**
		**Mean±SD**	**Min**	**Max**	**Male**	**Female**		
Unclassified	37	51.02±22.41	17	88	26	11	9	2
Dermatofibrosarcoma	22	48.72±14.78	25	74	14	8	12	2*
Rabdomyosarcoma	17	22.88±21.69	3	80	9	8	10	1
Leiomyosarcoma	10	49.50±18.04	19	86	8	2	5	2
Synovial sarcoma	6	36.00±9.59	23	50	4	2	3	1
Malignant peripheral nerve sheath tumor	5	44.20±20.77	23	71	2	3	3	-
Angiosarcoma	5	59.20±9.33	47	73	4	1	1	-
Kaposi sarcoma	5	55.60±19.80	26	80	4	1	-	-
Malignant fibrous histiocytoma	5	55.60±21.37	28	79	1	4	3	-
Liposarcoma	3	42.66±22.36	17	58	3	-	-	-
Fibrosarcoma	3	56.33±2.51	54	59	1	2	1	-
Primitive neuroectodermal tumors	2	42.00±39.59	14	70	1	1	-	-
Olfactory neuroblastoma	1	17.00	-	-	1	-	-	-
Alveolar soft part sarcoma	1	67.00	-	-	-	1	-	-
Total	122				78	44	47	8

* Fibrosarcomatous change in dermatofibrosarcoma

*Hard tissue sarcoma*

Hard tissue sarcomas comprised 30.68% (n=54) of the sarcoma cases. Osteosarcoma was the most prevalent cancer in the HTS group (81.48%). The HTS was more prevalent among the patients who were in their third decades of life with a mean age of 36.22 years. The male to female ratio of this type of sarcoma was obtained as 0.86. The mandible (53.7%) was identified as the most frequently involved location, followed by the maxilla (25.92%) and sinonasal region (9.25%). One patient developed radiation-induced osteosarcoma following receiving adjuvant radiotherapy treatment for head and neck carcinoma. Furthermore, chondroblastic osteosarcoma (n=18) was the common type of osteosarcoma subgroup (Table.3). 

**Table 3:** Distribution of primary hard tissue sarcoma cases based on age, gender, recurrence and metastasis (n=54)

**Histopathologic type**	**Number of cases**	**Age**			**Gender**		**Recurrence (n)**	**Metastasis (n)**
		**Mean±SD**	**Min**	**Max**	**Male**	**Female**		
Osteosarcoma	44	35.72±17.71	13	79	20	24	7	-
Chondrosarcoma	6	46.50±22.65	21	84	3	3	2	-
Ewing sarcoma	4	20.00±3.91	16	25	2	2	1	-
Total	54				25	29	10	-

*Sarcoma in children and adolescents*

There were 17 pediatric and adolescent patients under the age of 18 years in the cohort with a mean age of 13 years and male to female ratio of 1.42, representing 9.6% of all primary HNSs. In this age group, the oral cavity (47%) was the most commonly affected site. Furthermore, rhabdomyosarcoma and osteosarcoma were the typical microscopic variants in this group (Table.4).

**Table 4:** Distribution of pediatric hard tissue sarcoma cases based on age, gender, and involved location (n=17)

**Histopathologic type**	**Number of cases**	**Mean±SD**	**location**				**Gender**	
			**Oral cavity**	**Sinonasal region**	**Face**	**Neck**	**Male**	**Female**
Rhabdomyosarcoma	7	8.850±5.04	4	-	2	1	3	4
Osteosarcoma	5	15.40±2.40	3	2	-	-	2	3
Ewing sarcoma/PNET	2	15.00±1.41	1	-	1	-	2	-
Unclassified sarcoma	2	17.50±0.70	-	1	1	-	2	-
Olfactory neuroblastoma	1	17.00	-	1	-	-	1	-
Total	17		8	4	4	1	10	7

PNET: primitive neuroectodermal tumors

## Discussion

Epidemiological studies have demonstrated diverse results about the prevalence of various sarcomas in different countries (3,6,7).

Additionally, due to the rarity of HNSs, most of the published series have a rather small sample size (1,8). Therefore, it seems that complementary information about the various features of HNSs could be useful for clinicians and further research.

In the current study, HNSs accounted for 4.7 % of all body sarcomas. They occurred in all age groups and had a male predilection. These findings are similar to those reported in several studies (2,3,5). In our research, osteosarcoma was the most common HNS subtype. In a number of studies, osteosarcoma has been reported as the most common variant of HNSs (2,3); however, Stavrakas et al. found chondrosarcoma as the most common sarcoma (9). In the current research, the oral cavity was the most frequently affected site, which is in line with the results obtained by Alishahi (3). In other studies, sinonasal region, maxillary sinuses, and face were reported as the commonly involved locations (1,2,9).

In this cohort, STSs constituted 69.31% of all primary HNSs, which is consistent with the findings of other studies (1,2,5,10,11). On the contrary, Vassiliou et al and Alishahi et al. reported HTSs as the most common sarcomas with the frequencies of 57.78% and 54.27%, respectively (3,6). In a number of studies, rhabdomyosarcoma and unclassified sarcoma were presented as the most common STSs (6,12,13). It was believed that the epidemiological characteristics of different types of sarcomas vary among countries (3). In the current research, the most common STS was unclassified sarcoma (21%) in which further histological typing was not possible even with immunohistochemical procedures. Despite the improvements in immuno histochemistry and molecular markers, the categorization of sarcomas is still a challenge; accordingly, about 20% of all sarcomas are unclassified (1). 

Dermatofibrosarcoma was the second most frequent STS, which is in agreement with the findings of Huber et al. and Liuzzi et al. (14,15). Additionally, rhabdomyosarcoma was the third most frequent STSs. These different results may be related to geographical variations (3). For instance, in a study carried out in Africa, Kaposi’s sarcoma was the most common HNS relevant to HIV infections (16). Furthermore, in a study performed in Australia, malignant fibrous histiocytoma (MFH) was the most frequent histologic type, suggesting *ultraviolet* as one of the etiologic factors (5). Various studies have introduced MFH as the most common type of STS (2,5,11); nonetheless, the histopathological terminology for MFH has been changed, and the 2009 World Health Organization classification of STSs no longer retains the term MFH (5). 

Many studies similar to our research revealed that STS has a higher tendency toward affecting males (1,6,10-13). Our results revealed that the face, scalp, and neck were the commonly affected locations, which is in congruence with the results of several studies (1,10). Gorsky et al. reported the nasopharyngeal site as the most common location affected by STSs (13). However, based on the evidence, salivary gland sarcomas are rare with an incidence range of 0.3-1.5% (17). In the current study, about 1.6% of the STSs occurred in the salivary gland region. In our research population, the most frequent variant of HTS was osteosarcoma, followed by chondrosarcoma, which is in agreement with the findings of previous reports (3,2,18).The prevalence of chondrosarcoma was reported to be higher than that of osteosarcoma in a number of studies (5,9,19). In the present study, HTSs had a higher female predilection. Furthermore, the HTS patients had a lower mean age, compared to the STS group. Similar to our finding, Guevara-Canales et al. revealed a female predilection (20). However, some studies showed male tendency or no gender predilection (2,5,6). 

Tejani et al. also mentioned that the median age in HTS group was much lower than that in the STS group (10). The mandible was the most commonly involved site of occurrence, which is similar to the results of many previous reports (3,6,10,19,21). However, Barosa et al. reported the maxillary sinus as the most frequently affected location (2). In the current research, the recurrence and metastasis rates were obtained as 32.38% and 5%, respectively. These rates have been reported as 22-54% and 18-36% in other studies, respectively (1,7,10,12). It is worth mentioning that 9.6% of sarcomas developed under the age of 18 years with a mean age of 13 years. There was a male predilection (male to female ratio: 1.42) in this age range, and the oral cavity was the most commonly affected site. Similar to other studies, rhabdomyosarcoma and osteosarcoma were identified as the most prevalent types of sarcoma in this age group (1,3,22).

## Conclusion

The patterns of sarcoma affecting the head and neck regions regarding the site of involvement, rate of recurrence, age, and gender, obtained in this study were similar to those frequently found in other countries. Eventually, the implementation of large series studies, such as the current research, would contribute to the recognition of the precise prevalence of this disease and its microscopic variants, as well as the demographic data affecting this malignancy, thereby facilitating more accurate diagnosis and better treatment. 
